# Data for β-lactoglobulin conformational analysis after (-)-epigallocatechin gallate and metal ions binding

**DOI:** 10.1016/j.dib.2016.12.021

**Published:** 2016-12-21

**Authors:** Liangliang Zhang, Indra Dev Sahu, Man Xu, Yongmei Wang, Xinyu Hu

**Affiliations:** a Key Lab. of Biomass Energy and Material, Jiangsu Province; Institute of Chemical Industry of Forest Products, CAF, Nanjing 210042, China; b Chemistry and Biochemistry Department, Miami University, Oxford, OH 45056, USA

## Abstract

This data article contains complementary results related to the paper “Effect of metal ions on the binding reaction of (-)-epigallocatechin gallate to *β*-lactoglobulin” (Zhang et al., 2017) [1]. Data was obtained by circular dichroism (CD) spectroscopy to investigate potential *β*-lactoglobulin (*β*-Lg) conformational changes with different concentrations of EGCg and Cu^2+^ or Al^3+^ added to *β*-Lg. 500 µL of the 25 µM *β*-Lg solution containing EGCg (25 µM) or metal ions (0–500 µM) were measured, and the spectra were recorded. CD spectroscopy data present in this article indicated that the *β*-Lg-Cu, *β*-Lg-Al and *β*-Lg-EGCg interaction resulted in unfolding of the secondary structure of *β*-Lg.

**Specifications Table**

**Table:** 

Subject area	*Chemistry*
More specific subject area	*Polyphenol chemistry*
Type of data	*Figure*
How data was acquired	*MOS-500 spectropolarimeter (Bio-Logic, France)*
Data format	*Analyzed*
Experimental factors	*CD spectroscopy was performed with the method of* Li et al. [2].
Experimental features	*All samples were prepared in 20 mM PBS buffer at pH 7.4. 500 µL of the 25 µM β-Lg solution containing EGCg (25 µM) or metal ions (0–500 µM) were measured, and the spectra were recorded.*
Data source location	*Nanjing, China*
Data accessibility	*Data is with this article*

**Value of the data**

• The data provides some additional data on the effects of metal ions on the binding reaction of EGCg to *β*-Lg.
• The data indicated the conformational change of *β*-Lg after binding with EGCg or metal ions Cu, Al.
• The interaction between [*β*-Lg-Cu] and [*β*-Lg-Al] results in unfolding of the secondary structure of *β*-Lg.
• This data provide insights in understanding the effects of metal ions on the binding reaction of polyphenol compounds to *β*-Lg.

## 1 Data

Fig. 1 reports the CD spectra of *β*-Lg with different concentrations of EGCg or Cu^2+^ or Al^3+^. The negative bands at 222 nm could indicate the *α*-helix structure of the proteins [1,3].

## 2 Experimental design, materials and methods

### 2.1 Materials

EGCg (≥95%) and *β*-Lg (A variant, purity ≥90%) were purchased from Sigma-Aldrich Co. (St. Louis, MO, USA). Working solutions of EGCg (0.25 mM) were prepared by dissolving the EGCg in a 50% methanol solution. The working solution of *β*-Lg (25 µM) was prepared in 20 mM PBS buffer, pH 7.4 and stored in a refrigerator prior to use. The *β*-Lg and EGCg concentrations were determined spectrophotometrically by their extinction coefficients: *ε*_280_(*β*-Lg)=17600 M^−1^ cm^−1^ and *ε*_280_(EGCg)=9700 M^−1^ cm^−1^ at 280 nm [4], [5]. For *in vitro* experiments, the working solutions of Cu^2+^ and Al^3+^ (1.0 mM) were prepared by dissolving CuCl_2_·2H_2_O and AlCl_3_, respectively, in double-distilled water containing 0.1 M HCl to facilitate dissolution. All other reagents and solvents were of analytical reagent grade and used without further purification. All aqueous solutions were prepared using freshly double-distilled water.

### 2.2 Experimental design

CD spectroscopy was performed using a MOS-500 spectropolarimeter (Bio-Logic, France) with the modified method of Li et al. [2]. The CD spectra of the *β*-Lg, [*β*-Lg-EGCg] and [*β*-Lg-metal] systems were recorded between 190 and 250 nm by scanning the spectrum at 25 °C, with a scanning speed of 100 nm min^−1^, 2 s response time, and 1.0 nm step size. All samples were prepared in 20 mM PBS buffer at pH 7.4. To investigate the effect of EGCg, Cu^2+^ and Al^3+^ on the secondary structure of *β*-Lg, 500 µL of the 25 µM *β*-Lg solution containing EGCg (25 µM) or metal ions (0–500 µM) were measured, and the spectra were recorded. The samples were loaded into a rectangular quartz cuvette with a path length of 1 mm. The spectra of three consecutive scans were averaged and corrected by subtracting the solvent/buffer spectra.

## Figures and Tables

**Fig. S1:**
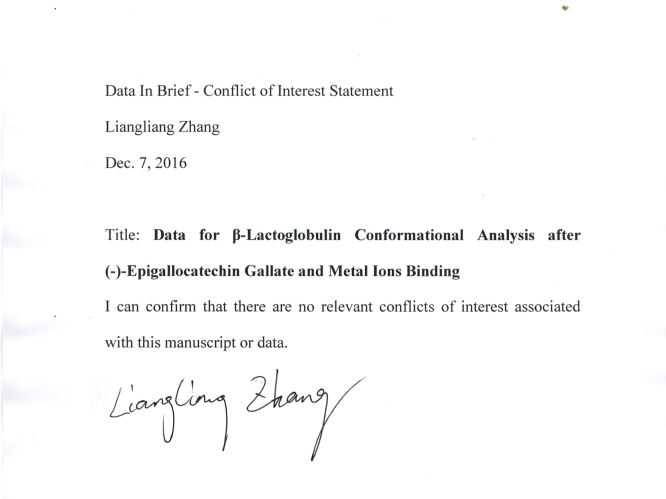


**Fig. 1:**
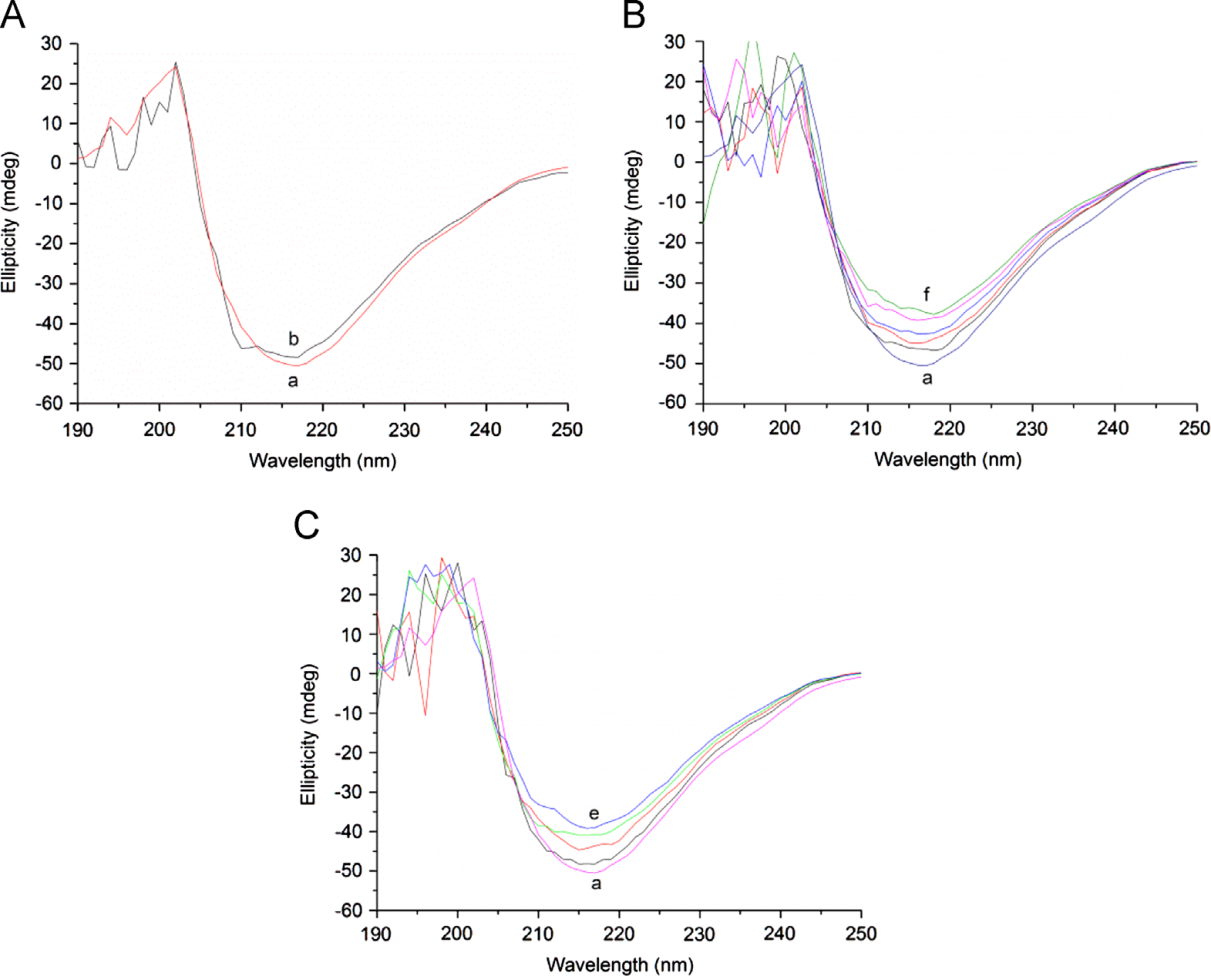
(A) CD spectra of *β*-Lg-EGCg system. a, (25 µM *β*-Lg), b (25 µM EGCg); (B) CD spectra of *β*-Lg-Cu system. a, (25 µM *β*-Lg), *c*(Cu^2+^): a (0), b (100 µM), c (200 µM), d (300 µM), e (400 µM), f (500 µM); (C) CD spectra of *β*-Lg-Al system. a (25 µM *β*-Lg), *c*(Al^3+^): a (0), b (100 µM), c (200 µM), d (300 µM), e (400 µM).
